# Nutritional habits and free grazing regimen of productive animals along with specific ingredients are influential factors for the antioxidant properties of milk: From farm to market

**DOI:** 10.3892/br.2020.1361

**Published:** 2020-09-29

**Authors:** Aristidis S. Veskoukis, Efthalia Kerasioti, Konstantinos Sidiropoulos, Ilektra Maragou, Zoi Skaperda, Demetrios Kouretas

Biomed Rep 13:31–36, 2020; DOI: 10.3892/br.2020.1301

Following the publication of this article, the authors have realized that they erroneously overlooked including details of the co-funding that they received for their research in the Declarations section of their paper. The complete information in the Funding section should have been given as follows (the added information is highlighted in bold):

**Funding**

The present study was funded by the post-graduate programs ‘Toxicology’ and ‘Biotechnology-Quality Assessment in Nutrition and the Environment’ in the Department of Biochemistry and Biotechnology at the University of Thessaly. **Co-financed by the European Regional Development Fund of the European Union and Greek national funds through the Operational Program Competitiveness, Entrepreneurship and Innovation, under the call RESEARCH - CREATE - INNOVATE (project code: ?1EDK-05479)**

The authors apologize to the co-funders of this study, and to the readership of the Journal for any inconvenience caused.

